# Selective release of muscle-specific, extracellular microRNAs during myogenic differentiation

**DOI:** 10.1093/hmg/ddw237

**Published:** 2016-07-27

**Authors:** Anna M.L. Coenen-Stass, Corinne A. Betts, Yi F. Lee, Imre Mäger, Mikko P. Turunen, Samir EL Andaloussi, Jennifer E. Morgan, Matthew J.A. Wood, Thomas C. Roberts

**Affiliations:** 1 Department of Physiology, Anatomy and Genetics, University of Oxford, South Parks Road, Oxford OX1 3QX, UK; 2 Department of Laboratory Medicine, Karolinska Institutet, Stockholm SE-141 57, Sweden; 3 Institute of Technology, University of Tartu, Nooruse 1, 50411 Tartu, Estonia; 4 Department of Biotechnology and Molecular Medicine, A.I. Virtanen Institute, University of Eastern Finland, 70150 Kuopio, Finland; 5 The Dubowitz Neuromuscular Centre, Molecular Neurosciences Section, Developmental Neurosciences Programme, UCL Institute of Child Health, London WC1N 1EH, UK; 6 Sanford Burnham Prebys Medical Discovery Institute, Development, Aging and Regeneration Program, La Jolla, CA 92037, USA

## Abstract

MyomiRs are muscle-specific microRNAs (miRNAs) that regulate myoblast proliferation and differentiation. Extracellular myomiRs (ex-myomiRs) are highly enriched in the serum of Duchenne Muscular Dystrophy (DMD) patients and dystrophic mouse models and consequently have potential as disease biomarkers. The biological significance of miRNAs present in the extracellular space is not currently well understood. Here we demonstrate that ex-myomiR levels are elevated in perinatal muscle development, during the regenerative phase that follows exercise-induced myoinjury, and concomitant with myoblast differentiation in culture. Whereas ex-myomiRs are progressively and specifically released by differentiating human primary myoblasts and C2C12 cultures, chemical induction of apoptosis in C2C12 cells results in indiscriminate miRNA release. The selective release of myomiRs as a consequence of cellular differentiation argues against the idea that they are solely waste products of muscle breakdown, and suggests they may serve a biological function in specific physiological contexts. Ex-myomiRs in culture supernatant and serum are predominantly non-vesicular, and their release is independent of ceramide-mediated vesicle secretion. Furthermore, ex-myomiRs levels are reduced in aged dystrophic mice, likely as a consequence of chronic muscle wasting. In conclusion, we show that myomiR release accompanies periods of myogenic differentiation in cell culture and *in vivo.* Serum myomiR abundance is therefore a function of the regenerative/degenerative status of the muscle, overall muscle mass, and tissue expression levels. These findings have implications for the use of ex-myomiRs as biomarkers for DMD disease progression and monitoring response to therapy.

## Introduction

MicroRNAs (miRNAs) are a class of small (21–22 nt), non-coding RNAs which regulate gene expression at the post-transcriptional level based on partial sequence complementarity (1). The majority of mammalian mRNA transcripts are subjected to miRNA-mediated regulation, and as such miRNAs are involved in the control of a wide variety of physiological processes including development, proliferation, differentiation, apoptosis, and myogenesis (2,3). Additionally, miRNAs have been implicated in various pathologies including oncogenesis, cardiovascular disease and immunological disorders (4).

Recently, miRNAs were found to be abundant and stable in biofluids including blood serum/plasma, cerebrospinal fluid (CSF), urine, milk, and saliva (5). Interestingly, altered profiles of extracellular miRNA (ex-miRNA) are associated with distinct physiological states (e.g. pregnancy (6)), and disease conditions, including myocardial infarction (7), muscle injury (8), liver damage (9), diabetes (10), and cancer (11). As a result, ex-miRNAs are promising disease biomarkers. An additional intriguing possibility is that miRNAs are selectively secreted, taken up by recipient cells and subsequently influence gene expression in a sequence-dependent manner. Currently, the cellular machinery that is involved in the packaging, release, and uptake of small RNAs is not well understood. Recent studies have identified a variety of carriers for ex-miRNAs including exosomes and microvesicles (12,13), apoptotic bodies (14), and vesicle-free lipoprotein (15,16) or protein complexes (17,18) that could account for their remarkable stability in the nuclease-rich extracellular environment.

Duchenne Muscular Dystrophy (DMD) is a progressive muscle wasting disorder that is caused by loss-of-function mutations in the *DMD* locus on the X-chromosome (19). The absence of dystrophin protein destabilises the sarcolemma, leading to repeated cycles of muscle degeneration and regeneration, accompanied by chronic inflammation and progressive fibrosis (20). We (21,22) and others (8,23) have identified a set of miRNAs (miR-1, miR-133 and miR-206) that are highly enriched in the circulation of dystrophic animal models (e.g. *mdx* mouse and CXMD_J_ dog) and DMD patients (24) compared to sera from unaffected controls. Importantly, these ex-miRNAs are potential DMD biomarkers as they are restored towards wild-type levels in *mdx* mice after dystrophin restoration by exon skipping (21–23,25). Notably, miR-1, miR-133 and miR-206 are among the most abundant miRNA species in myocytes (compromising more than 25% of all miRNAs) (26) and are involved in the control of muscle homeostasis by coordinating both myoblast proliferation and differentiation (27,28). The expression of these miRNAs is highly specific to skeletal and cardiac muscle, and so they are commonly referred to as myomiRs (29,30). The tissue-specific origins of myomiRs, and the large changes in abundance observed for extracellular myomiRs (ex-myomiRs) in dystrophic serum (50–100 fold (21)), makes muscle an attractive model system in which to investigate ex-miRNA biology.

Previously we have observed an asymmetry in myomiR expression patterns between the musculature and the circulation in *mdx* mice when compared to wild-type animals (21), highlighting that the reason for the high abundance of circulating myomiRs cannot be explained by expression changes in muscle tissue alone. Alternatively, gross muscle breakdown and/or passive leakage due to dystrophic pathology-associated membrane defects may explain the presence of ex-myomiRs in the circulation. In this study, we show that ex-myomiRs can be selectively released in physiological contexts including postnatal development, following physical exercise, and during cellular differentiation. Our findings reveal fundamental new insights into ex-miRNA biology and have additional implications for the use of ex-myomiRs as disease biomarkers for DMD.

## Results

### MyomiR release during neonatal muscle development and growth

During the first four weeks of life, murine muscle mass increases rapidly (31). Postnatal muscle development consists of a very active phase of satellite cell proliferation and myogenic differentiation (32,33), accompanied by upregulation of intracellular myomiRs. We hypothesised that this development process may also result in an altered serum myomiR signature and sought to investigate ex-myomiR abundance during the first 28 postnatal days (PND) in wild-type (C57) and dystrophic *mdx* mice.

**Figure 1.**
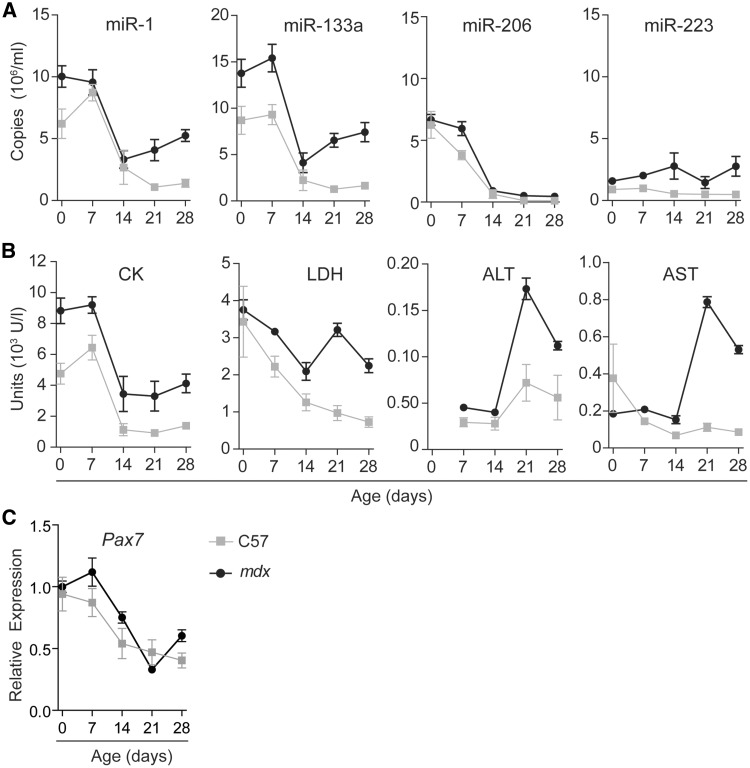
Levels of ex-myomiRs are elevated in juvenile wild-type and pre-symptomatic *mdx* mice. Wild-type (C57) and *mdx* mice (*n*=4-8) were sacrificed at the indicated time points and blood was assayed for (**A**) ex-miRNA levels and (**B**) clinical biochemistry markers (CK, LDH, AST and ALT). Ex-myomiR and miR-223 levels were measured by RT-qPCR using synthetic RNA standards to determine absolute copy numbers. (**C**) *Pax7* transcript levels were determined in the TA muscle by RT-qPCR and normalised to TATA Box Binding Protein (*Tbp*) expression. Values are mean +/- SEM. Two-way ANOVA with Bonferroni post testing was performed and *P*-values for interaction, group and time factors are listed in Supplementary Material, Table S1.

Ex-myomiRs were highly enriched in the circulation at birth and throughout the first week of life in both genotypes before declining until PND 14 and then rising gradually for *mdx* mice from PND 21 (Figure 1A). According to two-way ANOVA (Supplementary Material, Table S1), the level of each individual released myomiR changed significantly with time (*P*-value of the time factor *P *<* *0.0001 for all myomiRs). Most importantly, miR-1, miR-133a and miR-206 dynamics followed a similar pattern in both mouse strains (*P*-value of interaction factor *P > *0.05 for all myomiRs, i.e. not significant). We used the granulocyte-specific miR-223 as a non-myomiR control miRNA since it is stably abundant in murine serum (both wild-type and *mdx*) and has therefore previously been used as an endogenous control by our group (22,34) and others (23,25). Notably, serum abundance of miR-223 remained unchanged over the time period investigated (non-significant time factor).

In parallel, we measured clinical biochemistry markers of tissue damage that have been shown to be elevated in DMD, including creatine kinase (CK), alanine aminotransferase (ALT), aspartate aminotransferase (AST) and lactate dehydrogenase (LDH) (35). In contrast to CK and the myomiRs, analysis of LDH, ALT and AST showed a noticeable peak at PND 21 and levels remained elevated thereafter, consistent with the known onset of dystrophic pathology in *mdx* mice at three weeks of age (36,37). (Figure 1B, Supplementary Material, Table S1). Interestingly, serum CK levels closely followed the pattern observed for myomiRs (Figure 1B). These findings are consistent with observations of elevated CK in healthy new-born humans that return to normal within the first 10 days of life (38). In summary, the release of ex-myomiRs in asymptomatic and pre-symptomatic mice follows a similar pattern, with a large increase in abundancy during the perinatal phase which occurs in the absence of elevated AST and ALT levels.

We further monitored *Pax7* transcript levels in the tibialis anterior (TA) muscles to confirm satellite cell proliferation (Figure 1C). In both genotypes, *Pax7* was highly abundant during the first week of life and expression subsequently dropped to a low baseline level at 21 PND. *mdx* mice exhibited a slight increase in *Pax7* expression at 4 weeks of age, possibly correlated to muscle turn-over caused by the onset of pathology.

### Exercise induces delayed myomiR release

Given the dynamic changes in ex-myomiR abundance during postnatal muscle development, we were interested to further investigate a possible correlation between muscle regeneration in adult mice and ex-myomiR release. Therefore, we used physical exercise to induce regeneration through low-level, body-wide, muscle injury. Adult muscle regeneration after injury is reminiscent of embryonic myogenesis and consists of two interdependent phases (39). Initially, myofiber necrosis and inflammation is observed shortly after injury. This is followed by activation of satellite cells that initiate regeneration, remodeling and functional repair. Typically, satellite cells are activated 3 days after muscle injury, fuse with myotubes after 5 days and begin to close the injury lesion 7 days after the initial muscle damage (40).

**Figure 2.**
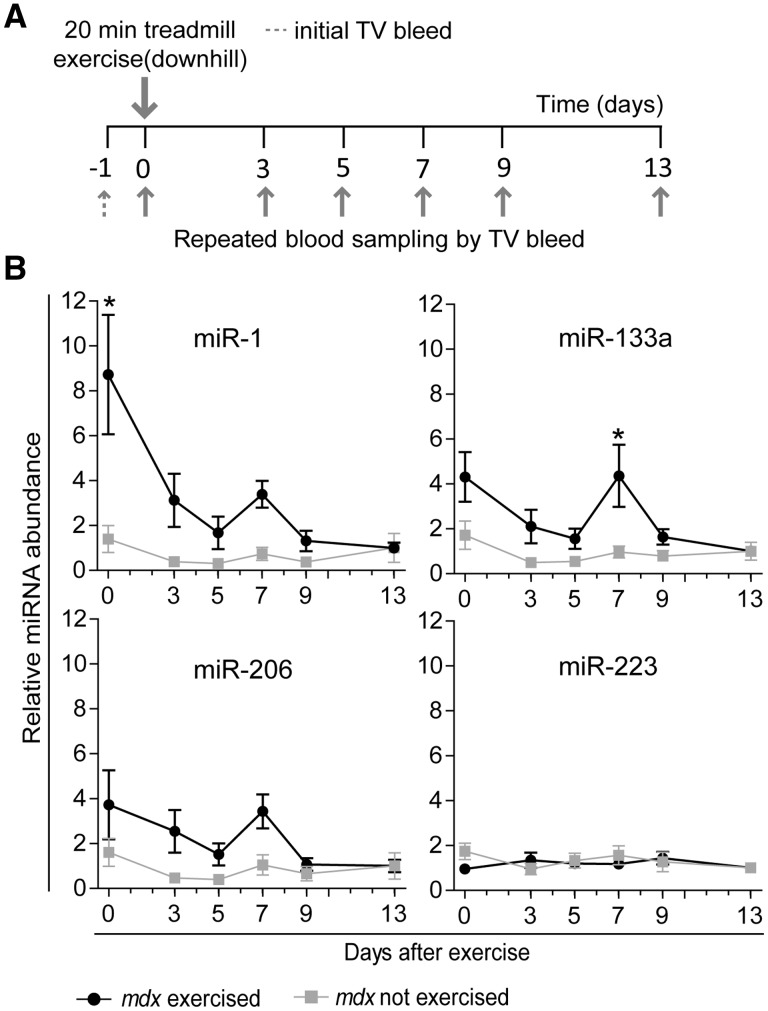
Acute eccentric exercise induces a biphasic abundance pattern of myomiR release in *mdx* mice. (**A**) 12 week old male *mdx* mice (*n*=8) were exercised by running downhill for 20 min at increasing speed on a treadmill with a 15˚ incline and compared with unexercised controls (*n*=5). Blood was collected at indicated time points by serially sampling from each mouse. (**B**) Levels of miRNAs in serum were determined by RT-qPCR. Expression of myomiRs was normalised to the day -1 bleed for each individual animal respectively to minimise noise due to inter-animal variability. All values are mean +/- SEM. *P<0.05 (Two-way ANOVA, Bonferroni post test). *P*-values for interaction, group and time factors are listed in Supplementary Material, Table S2.

Here, we subjected *mdx* mice to 20 min of downhill exercise and harvested blood samples repeatedly, every 2-4 days until 13 days after exercise in order to determine any changes in serum myomiR abundance that could result from either immediate muscle injury or a delayed regenerative response (Figure 2A). Repeated sampling via the tail vein and normalisation to day -1 bleeds for each individual mouse was employed in order to reduce noise caused by inter-animal variability. *mdx* mice were used as their serum is highly enriched for ex-myomiRs (21) meaning that ex-myomiR detection is technically facile. To further exacerbate exercise-mediated myoinjury, we initially subjected the mice to downhill exercise, as eccentric contraction is known to cause more muscle damage than other types of contractions (41) and *mdx* mice have been shown to be highly susceptible to stretch-induced muscle damage (42).

Interestingly, all circulating myomiRs exhibited a dynamic abundance pattern with an immediate increase (day 0) and a second peak of smaller magnitude at day 7 after exercise (Figure 2B). Importantly, this biphasic abundance pattern was unique to the exercised group and highly similar for all myomiRs. According to two-way ANOVA (Supplementary Material, Table S2), ex-myomiRs abundance changed significantly with time (*P*-value of time factor *P *<* *0.05 for all myomiRs). Furthermore, the exercised and unexercised group behaved significantly different for miR-1 and miR-133a (*P*-value of group factor *P *<* *0.05). Similarly, *post hoc* analysis demonstrated a significant increase in miR-1 abundance immediately after exercise and a significant increase in miR-133a abundance 7 days after exercise. For all myomiRs, levels returned to the unexercised baseline within 4 days following the second peak. Conversely, the non-myomiR control (miR-223) did not exhibit any significant changes throughout the duration of the experiment.

The exercise experiment was replicated in wild-type mice although in this case no increases in ex-myomiRs were observed, likely as a result of the reduced sensitivity of healthy muscles to contraction-induced damage relative to *mdx* (43,44) (Supplementary Materials, Figure S1 and Table S3). Previously, we have shown altered miRNA release in wild-type mice immediately after inducing localised myoinjury with cardiotoxin (CTX) in the TA muscle of wild-type mice (22). Here we reproduced this experiment with the methodological improvements described above (i.e. day -1 normalisation and serial measurements taken over an extended time period), to additionally monitor any delayed changes in ex-myomiR release due to muscle regeneration following injury. Similar to our previous findings, CTX injury caused an increase in ex-myomiRs immediately after the treatment (Supplementary Materials, Figure S2 and Table S4). However, we could not detect any significant changes at later time points, possibly because injury followed by regeneration in a single muscle is insufficient to increase ex-myomiRs to a detectable level beyond the initial degenerative phase. Furthermore, it is important to note that myomiR abundance in non-dystrophic mice is inherently low and close to the limit of detection, which complicates the measurement of relatively small changes in abundance compared to background levels.

**Figure 3.**
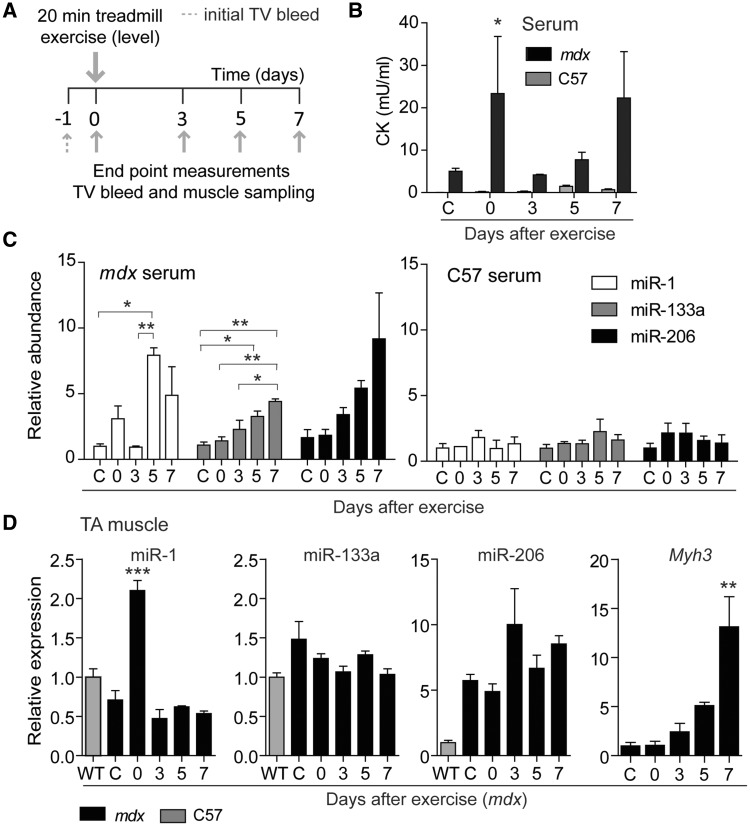
End point analysis of miRNAs and CK in serum and tissue after exercise. (**A**) 12-week-old male *mdx* and wild-type (C57) mice were exercised for 20 min by running on a level surface at increasing speed. Animals were sacrificed at the indicated end points (*n*=3) and tissue samples and serum harvested. (**B**) CK was measured in *mdx* and wild-type serum. Levels of miRNAs in (**C**) serum or muscle of *mdx* and wild-type (**D** and Supplementary Material, Figure S1) were determined by RT-qPCR. *Myh3* transcript levels were measured by RT-qPCR and normalised to TATA Box Binding Protein (*Tbp*) expression. *Myh3* was undetectable in wild-type mice (D). CK= Creatine Kinase, WT= wild-type, C= not exercised control animals. All values are mean + SEM. *P<0.05, **P<0.01, ***P<0.001 (Two-way ANOVA with Bonferroni post test (B) (*P*-values are listed in Supplementary Material, Table S5) and one-way ANOVA with Bonferroni post test (C,D).

Next, we exercised *mdx* and wild-type mice with a slightly altered exercise protocol without downhill incline with the aim of decreasing the severity of the initial myoinjury and observing whether the levels of myomiRs released immediately after exercise similarly decrease (Figure 3A). Conversely, we were also interested to see if the delayed increases in myomiR abundance could still be detected with this modified exercise protocol. Additionally, we performed end point measurements to assess myomiR expression in skeletal muscle, and to allow for sampling of larger volumes of serum and analysis of serum CK in parallel.

CK was significantly elevated in the circulation of *mdx* mice compared to wild-type as reported previously (45) (Figure 3B, Supplementary Material, Table S5). However, CK levels after exercise varied considerably across animals, consistent with other studies (46). Interestingly, no significant increases in ex-myomiR levels were observed immediately after treadmill running on a level surface (Figure 3C). Therefore, serum CK levels were more affected by exercise than were ex-myomiRs, which is in agreement with previous studies (8), although only if measured immediately after exercise activity. Importantly, miR-1 exhibited a similar increase in abundance 5 days after exercise as observed in the downhill exercise experiment (Figure 2), possibly coupled to the regenerative phase. Likewise, miR-133a levels increased progressively following exercise and miR-206 exhibited a very similar pattern but failed to reach statistical significance at the *P *<* *0.05 level due to high inter-replicate variation. As with the previous exercise study, we were unable to detect any changes in ex-myomiR abundance in wild-type animals for either serum or skeletal muscle (Figure 3, Supplementary Figure S1).

In contrast, analysis of miRNA levels in *mdx* TA muscle sections harvested at the same time as the serum samples showed upregulation of miR-1 expression immediately after exercise, but downregulation at later time points (Figure 3C). miR-206 expression was increased in *mdx* TA muscle relative to wild-type animals, as described previously (21) but expression was not altered after exercise. No changes in miR-133a were observed in TA between wild-type and *mdx*, or following exercise thus highlighting that expression levels in tissue and abundance in serum do not always correlate (21). To confirm that physical exercised induced regeneration, we measured the transcript levels of myosin heavy chain 3 (*Mhy3*) in the TA muscle. Typically, *Mhy*3 is expressed during embryonic and fetal muscle development, but is also re-expressed in regenerating fibres after injury (47). In *mdx* mice *Mhy3* levels were significantly increased 7 days after exercise (Figure 3C) whereas we were unable to detect the transcript in wild-type muscle at any time point, thus confirming that the exercise regimen was insufficient to induce regeneration in non-dystrophic mice. In summary, serum myomiRs in dystrophic *mdx* mice exhibit dynamic changes in abundance depending on the mode of exercise, possibly reflecting the degenerative and regenerative status of the muscle.

### MyomiR release is reduced in aged *mdx* mice

The natural process of muscle loss during aging is exacerbated in *mdx* mice due to repeated cycles of degeneration and regeneration that follow as a consequence of dystrophin-deficiency (48). In particular, at 72 weeks of age, the hind limb muscles of *mdx* mice were shown to decrease in muscle mass by more than 50% in TA, extensor digitorum longus, soleus, and plantaris, and by up to 70% in quadriceps and gastrocnemius, compared to age-matched wild-type mice (49). Here, we were motivated to determine whether chronic muscle-wasting in aged *mdx* mice is reflected in serum myomiR abundance. To this end, we compared the levels of myomiRs and clinical chemistry biomarkers in the circulation of adult (14 weeks) and aged (88 weeks) *mdx* and wild-type animals.

**Figure 4.**
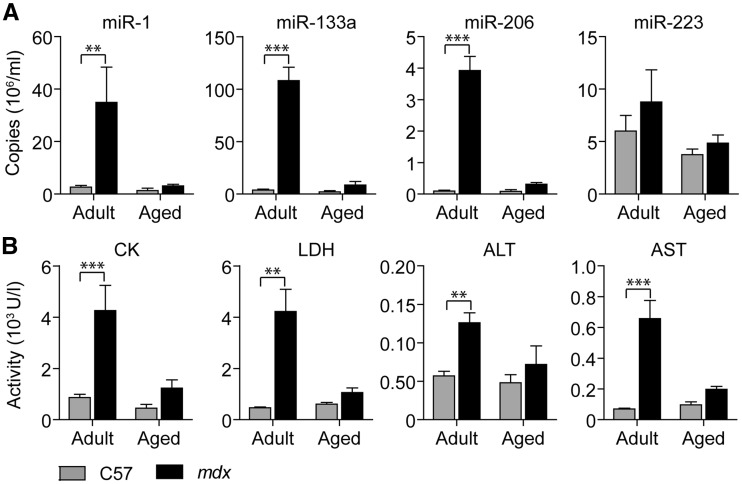
Serum myomiRs and clinical biochemistry markers are decreased in aged *mdx* mice. Blood from adult (14-week old) and aged (88-week old) wild-type (C57) and *mdx* mice (*n *=* *4–5) was harvested and assayed for (**A**) miRNA abundance and (**B**) clinical biochemistry parameters. (A) Serum myomiR levels were measured using RT-qPCR with synthetic RNA standards for absolute quantification. (B) In parallel, clinical biochemistry parameters were analysed using standard enzymatic assays. All values are mean + SEM. **P <* *0.01, ***P <* *0.001, (Two-way ANOVA, Bonferroni post test). *P*-values for interaction, group and time factors are listed in Supplementary Material, Table S6.

Consistent with previous data, both ex-myomiR levels and clinical biochemistry markers of tissue injury were significantly increased in adult *mdx* compared to adult wild-type mice (21,35) (Figure 4 and Supplementary Material, Table S6). Ex-myomiRs exhibited larger fold changes at 14 weeks (ranging from 13- to 42-fold) than CK, LDH, ALT and AST (ranging from 2- to 9-fold). Conversely, neither the ex-myomiRs nor clinical biochemistry serum markers were significantly different between genotypes in aged mice. Ex-myomiR abundance was restored to near wild-type levels in the aged mice, likely as a result of aging-associated muscle loss. In agreement, the body weight of aged *mdx* mice was significantly decreased compared to aged wild-type mice (Supplementary Material, Figure S3A). Furthermore, Hematoxylin and Eosin staining of the quadriceps femoris muscles demonstrated severely advanced pathology in aged dystrophic mice with extensive fibrosis and adipose deposition (Supplementary Material, Figure S3B. Serum myomiR levels in aged wild-type mice were not significantly altered compared to adult non-dystrophic animals. Notably, levels of the non-myomiR control miR-223 were unaffected by age or dystrophic pathology. Given their muscle-specific origins, these data suggest that serum myomiR abundance is also correlated to muscle mass in addition to reflecting the regenerative/degenerative status of muscle.

### Progressive myomiR release during myogenic differentiation

**Figure 5.**
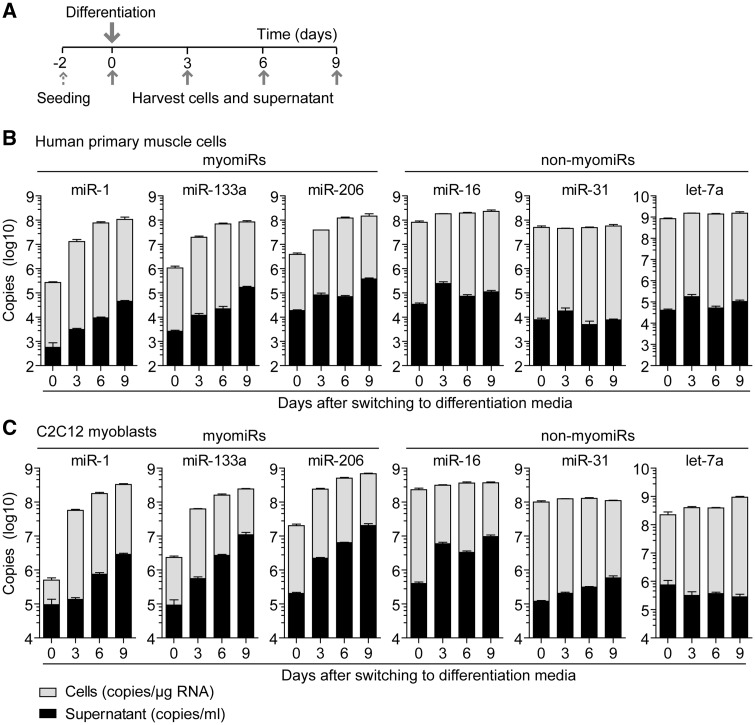
Progressive ex-myomiR release during myogenic differentiation in primary human and immortalised murine C2C12 myoblasts. Human and murine myoblasts were differentiated for up to 9 days in reduced serum media. (**A**) Cells and conditioned media were sampled as indicated. Levels of myomiR and non-myomiR control miRNAs in (**B**) human primary muscle cells and (**C**) C2C12 cells were determined by RT-qPCR using synthetic RNA standards for absolute quantification. Expression of cellular miRNAs was normalised to total RNA content (copies/µg RNA). The cell supernatant was sampled over 3 days periods and miRNA abundance reported as copies/ml. Values are mean + SEM, *n*=3–4.

Given that myogenic differentiation is accompanied by increased expression of cellular myomiRs, we were interested to investigate whether intracellular myomiR induction in muscle cell cultures is correlated with an elevated release of muscle-specific miRNAs, reminiscent of the myomiR release we observed *in vivo* during muscle development and regeneration. Firstly, primary human myoblasts were differentiated into multinucleated myotubes over the course of 9 days. RNA was isolated from cells and conditioned media collected every 3 days. The medium was replaced at regular intervals so that each supernatant sample represents the quantity of released miRNAs over the 3 day period and not the accumulation of secreted miRNA over the entire duration of differentiation (Figure 5A). Myotube formation was confirmed by light microscopy and quantification of transcripts of the myogenic regulatory factors *MYOD1* and *MYOG* (Supplementary Material, Figure S4).

Cellular expression of myomiRs was strongly and progressively induced with a 100-1000 fold increase in expression after 9 days of differentiation (Figure 5B). Absolute intracellular myomiR copy numbers after 9 days of differentiation reached similar levels of ∼100 million copies/µg RNA with miR-206 and miR-1 being the most abundant species measured. Concurrently, myogenic differentiation was associated with a strong, progressive increase (up to ∼100 fold) in the release of myomiRs, especially at the later stages of differentiation where multinucleated myotubes were visible (Supplementary Material, Figure S4C). Despite slightly differing patterns of release, after 9 days the absolute levels of myomiRs in the media were similar for miR-206 (0.4 million copies/ml) and miR-133a (0.2 million copies/ml) and somewhat lower for miR-1 (0.04 million copies/ml). Notably, cellular miRNA and secreted ex-miRNA levels were positively correlated for all myomiRs (Pearson coefficients: 0.7809 (miR-1), 0.7515 (miR-133a), 0.8219 (miR-206); all *P *<* *0.01, Supplementary Material, Table S7). Three additional non-myomiR control miRNAs were measured in parallel: miR-16 (ubiquitously expressed, previously used for miRNA normalisation in tissues (21,50)), let-7a (highly abundant in muscle tissues (51)), miR-31 (involved in regulating muscle processes (52) and recently proposed as a reference miRNA in serum (53)). All three control miRNAs exhibited relatively stable expression in cells, and stable abundance in cell culture supernatant over the course of the experiment (Figure 5B). Conversely, there was no significant correlation between cellular and secreted miRNA levels for these three non-myomiR controls. Importantly, let-7a was highly abundant (1.5 billion copies/µg RNA) in muscle cells throughout the period of differentiation. Considering this high-intracellular abundancy, let-7a was found at comparatively low levels in the supernatant (0.1 million copies/ml), thus highlighting the selectivity of myomiR release.

As primary muscle cells are of limited availability and are difficult to culture for extended periods of time, we were interested to see if these findings could be recapitulated in an immortalised myoblast cell line (C2C12). Upon serum depletion, C2C12 cells differentiated to form multinucleated myotubes with a concomitant induction of cellular myomiR expression (Figure 5C). Myogenic differentiation was visualised by immunostaining for Myosin Heavy Chain (MHC)-positive myotubes, which became apparent three days in differentiation conditions, and continued to mature into larger myotubes (Supplementary Material, Figure S4D). Importantly, myogenic differentiation progressively induced myomiR release up to ∼100 fold after 9 days of differentiation. As with the primary human myoblasts, cellular and secreted levels of myomiRs in C2C12 cells were likewise strongly correlated (Pearson coefficients: 0.9291 (miR-1), 0.8878 (miR-133a), 0.8937 (miR-206); all *P *<* *0.0001, Supplementary Material, Table S7) whereas the non-myomiR controls were not correlated (miR-31 and let7-a) or had weak correlation coefficients (miR-16). Notably, the absolute levels of myomiRs in the media were higher in C2C12 cells than in primary myoblast cells, probably due to higher cell density (∼2.5-fold more nuclei per image section, data not shown) that these cells achieve in culture.

**Figure 6.**
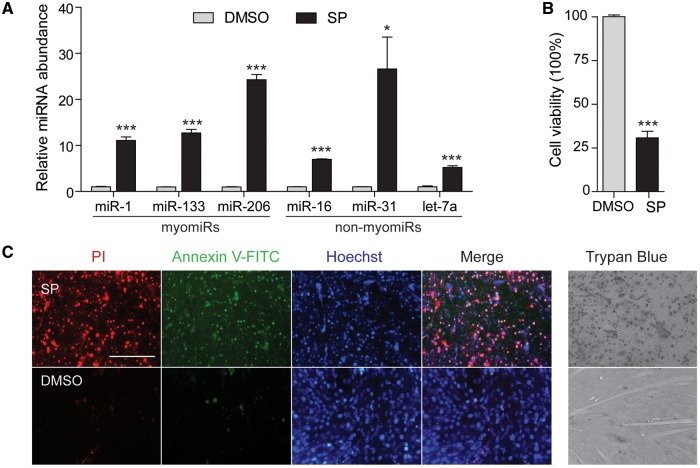
Induction of apoptosis in C2C12 myotubes induces indiscriminate ex-miRNA release. C2C12 cells were differentiated for 6 days and then apoptosis was induced by incubation with 1 µM Staurosporine (SP) for 24 h. (**A**) RNA was extracted from conditioned cell culture supernatants and miRNA levels were determined by RT-qPCR. Fold changes in miRNA abundance after SP treatment are shown relative to DMSO-treated control cultures. (**B**) Cell viability was determined by MTS assay and is reported as a percentage of the DMSO-treated control group. (**C**) Detection of apoptosis by Annexin V-FITC and Propodium Iodide (PI) fluorescence staining in Staurosporine treated (upper panel) and control cells (lower panel). Scale bar represents 200 µm (20x magnification). Apoptosis was further assessed using Trypan Blue staining to measure cell viability. All values are mean +SEM. *P<0.05, ***P<0.001 (unpaired *t*-test, 2 tailed, *n*=3).

Apoptosis is known to accompany normal myogenic differentiation, especially during the first few days after serum withdrawal (54). To ensure the progressive increase in ex-myomiRs described above was not due to passive release by apoptotic cells, cell viability was monitored throughout the course of differentiation using a Trypan Blue dye exclusion assay (Supplementary Material, Figure S4E). Inspection of culture wells revealed relatively few detached cells and only a minority of cells exhibited dye uptake. In addition, apoptosis was induced in differentiated C2C12 myotubes by treatment with Staurosporine (SP) and miRNA release was measured after 24 h. Both myomiRs and non-myomiRs were highly enriched (all *P *<* *0.001, with the exception of miR-31 (*P *<* *0.05)) in media from apoptotic cultures compared to vehicle treated cultures (Figure 6A). Release of myomiRs was increased by 10–25-fold in media after SP treatment. Non-myomiRs were similarly enriched in apoptotic media (5–25-fold), although miR-16 and let-7a were enriched to a lesser extent. Induction of widespread cell death was confirmed by MTS assay (Figure 6B) and fluorescence staining for Annexin V-FITC and propidium iodide (to label early-stage apoptosis and late-stage apoptosis/necrosis respectively) (Figure 6C). Given the dramatic increase in the myomiR release during normal differentiation (100–1000-fold relative to undifferentiated cells) it is unlikely that the non-selective release caused by apoptosis alone can account for ex-myomiR release in differentiating C2C12 culture media.

In summary, both cell models demonstrate that elevated levels of ex-myomiRs follow myogenic differentiation in healthy, non-dystrophic myoblasts, thus supporting the idea that ex-myomiRs are released under physiological conditions. Additionally, our findings demonstrate that absolute expression is not necessarily linked to the amount of miRNA release (as in the case of let-7a), consistent with the hypothesis of a sequence-selective mechanism of miRNA secretion. Finally, the ability to model ex-myomiR release in C2C12 myoblast cultures (with a homogenous cell population with sufficient capacity for both proliferation and differentiation) will be useful for further mechanistic studies.

### Mechanism of extracellular myomiR release and transport

Recently, a number of ex-miRNA carriers have been identified that are capable of protecting ex-miRNAs from degradation in the otherwise hostile, nuclease-rich environment of the blood. Studies that have compared the number of miRNAs found in exosomes and microvesicles (together referred to as extracellular vesicles (EVs)) with those miRNAs found outside of vesicles have concluded that only 1–5% of circulating of ex-miRNAs are associated with lipid-based carriers whereas the majority are stabilised in protein-complexes (15,17,18,55). Similarly, we have previously shown that serum myomiRs are predominantly non-vesicular, and co-precipitate with Argonaute-2 and Apolipoprotein-A1 (22). Importantly, the majority of studies have used ultracentrifugation (UC) to isolate EVs, a relatively crude methodology with significant limitations. UC has the disadvantage that it partly co-pellets large protein complexes, and centrifugation at high speed can cause EV aggregation, fusion and/or rupture (56). Here, we applied an improved methodology for EV isolation that combines ultrafiltration and size-exclusion-chromatography (UF-SEC) which enables the isolation of intact EVs with high purity and yield (57).

**Figure 7.**
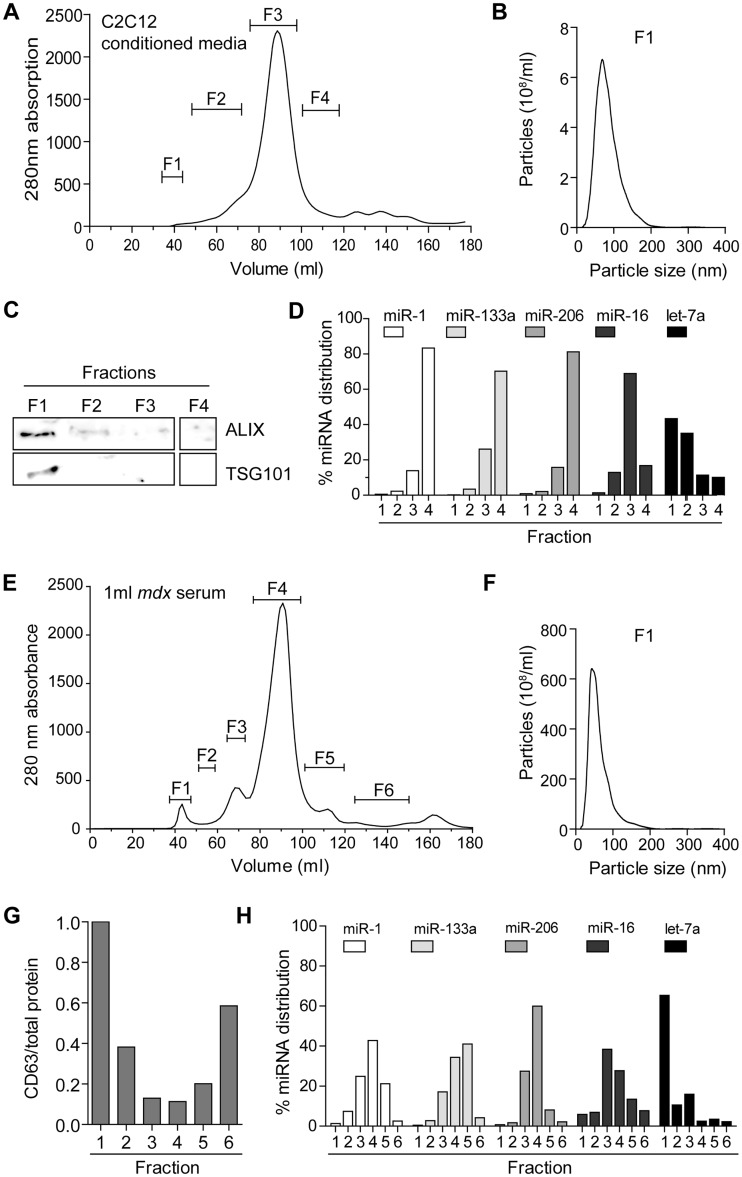
Ex-myomiRs in myotube-conditioned media and murine serum co-elute with protein complexes and are predominantly non-vesicular. 50 ml of conditioned media collected from differentiated C2C12 cells (A–D) and 1 ml of pooled serum from 12-week-old male *mdx* mice (E-H) was fractionated via UF-SEC. (**A**) The protein content was monitored using the 280 nm absorbance and eluates were pooled into 4 new fractions as indicated. (**B**) NTA analysis shows a modal particle size of ∼80 nm in F1. (**C**) Exosomal protein markers (ALIX and TSG101) were highly enriched in F1 as determined by Western blot analysis. (**D**) RNA was extracted from each pooled fraction and miRNA abundance measured by RT-qPCR. Values are depicted as the percentage distribution across all of the fractions. (**E**) Serum was fractionated and divided into six different fractions as indicated in the 280 nm absorbance trace. (**F**) NTA analysis shows a modal vesicle size of ∼65 nm in F1. (**G**) CD63 abundance was quantified using ELISA and normalised to total protein concentration to indicate fraction purity. (**H**) RNA was extracted from each fraction and miRNA abundance was quantified by RT-qPCR and normalised as described above.

As we have shown that differentiating C2C12 cells are suitable to model miRNAs release, we used this cell line to gain insight into ex-myomiR export and transport. During SEC of C2C12 cell culture supernatant, protein content was monitored by measuring the absorbance at 280 nm. Based on the absorbance trace and previous data, individual eluates from the SEC were pooled into four fractions (F1-4) (Figure 7A). Typically, EVs are contained in fraction F1 (57) and nanoparticle tracking analysis (NTA) determined the modal particle size to be ∼80 nm in this fraction (Figure 7B). The exosome marker proteins ALIX (PDC6IP) and TSG101 (58) were highly enriched in fraction F1 as determined by Western blot (Figure 7C). Together these findings confirm that EVs within fraction F1 exhibit typical exosomal properties. MyomiRs analysed in the pooled fractions showed a similar pattern of abundance with ∼80% of each miRNA detected in F4 and ∼20% in F3. Conversely, less than 1% could be detected in the F1 fraction, thus demonstrating that ex-myomiRs are predominantly present in small protein complexes, while only a small minority are associated with vesicles. In contrast, the non-myomiR controls miR-16 and let-7a exhibited different distribution patterns. In particular, let-7a was also found to be abundant in the vesicular fraction F1 (∼40%). These data are consistent with previous studies in human plasma which observed let-7a and miR-16 as exhibiting vesicular and non-vesicular distributions, respectively (17), and therefore underline the robustness of our UF-SEC fractionation methodology.

We next applied the UF-SEC technique to serum from *mdx* mice (Figure 7E–H). Notably, we observed an early protein peak in the 280 nm absorption trace corresponding to fraction F1. NTA analysis demonstrated that vesicles in F1 had a modal size of ∼65 nm. We were unable to detect exosomal protein markers via Western blot although CD63 antigen CD63 an exosome surface protein (58), was enriched in fraction F1 as determined by ELISA (Figure 7G). MyomiR abundance was measured in each fraction and, consistent with our cell culture findings, these miRNAs were found to be highly abundant in the fractions with high protein content. In contrast, ∼1% of myomiRs were found in the vesicular fraction F1, thus providing further evidence that C2C12 cultures faithfully model *in vivo* myomiR release.

**Figure 8.**
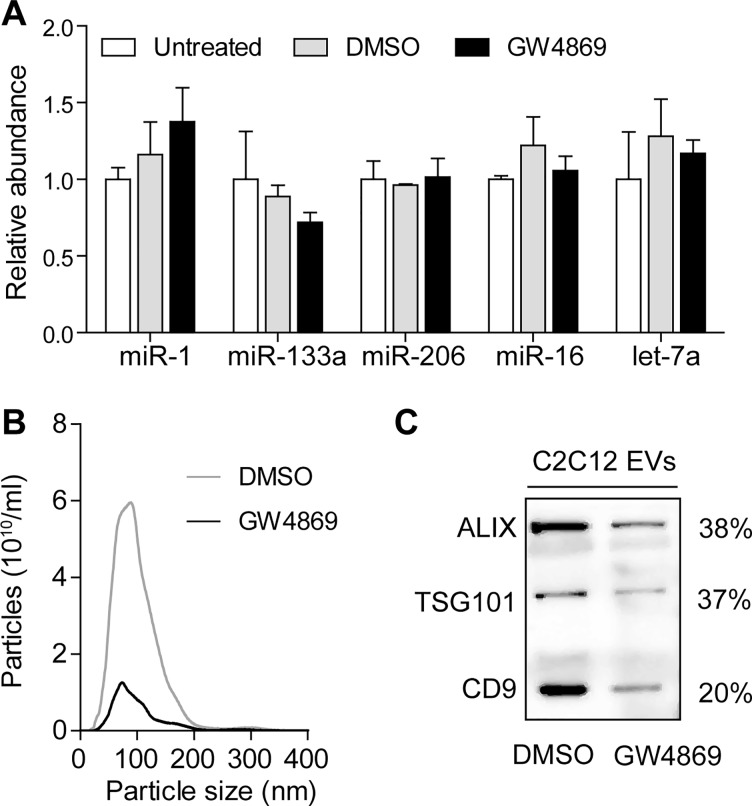
Inhibition of exosome secretion does not affect myomiR release in C2C12 myotubes. C2C12 cells were differentiated for 4 days and treated with 10 µM GW4869 to block exosome secretion via the ceramide pathway. (**A**) RNA was harvested from C2C12 myotube culture supernatants and miRNA levels measured by RT-qPCR. Mean values for the untreated group were returned to 1 for each miRNA. To confirm successful decrease in exosome secretion by the small molecule inhibitor, EVs were isolated from drug- and vehicle-treated cells. (**B**) Particle numbers were determined by NTA analysis in isolated EVs of GW4869 and DMSO treated cells. The modal size was ∼80 nm for both samples. (**C**) Western blot of the isolated EVs showed a decrease in exosomal marker proteins (ALIX, TSG101 and CD9) in GW4869 treated cells compared to vehicle treated cells (DMSO). Values are mean + SEM.

It is currently unclear how non-vesicular ex-miRNAs can cross the plasma membrane and be released into the extracellular space since a transmembrane export mechanism for non-lipid-based carriers has not yet been described. One possibility is that protein-bound miRNAs are initially packaged into vesicles which are released into the extracellular space and are subsequently degraded to liberate their miRNA-protein cargos. Consequently, we used GW4869, an inhibitor of neutral sphingomyelinase 2 (nSMase2) to block exosome secretion via the ceramide pathway in differentiated C2C12 cells and then monitored the effect on myomiR release. Interestingly, no significant reduction in myomiR abundance was measured when analysing the supernatant of GW4869-treated cells compared with DMSO-treated or untreated cells (Figure 8A). To confirm that the GW4869 treatment successfully down-regulated exosome secretion, we isolated EVs from C2C12 myotube conditioned media. Notably, exosome marker proteins were decreased in exosome preparations isolated from GW4869-treated cells by 60-80% as demonstrated by Western blot (Figure 8B). Additionally, NTA showed a ∼6 fold decrease in particle numbers after GW4869 treatment, thus confirming the successful inhibition of exosome secretion (Figure 8C). Given that we did not observe any reduction in myomiR release in unfractionated conditioned media, we conclude that EV-mediated secretion is unlikely to be responsible for the majority of myomiR release.

## Discussions

While muscle pathology clearly contributes to an enrichment of muscle-specific miRNAs in the circulation, here we show that, the release process can also be dynamic and myomiR-specific in various physiological contexts, including during postnatal muscle development, after physical exercise, and as myoblasts differentiate in culture. As a result, we conclude that myomiR release accompanies periods of myogenic differentiation and serum myomiR abundance is likely to be a function of muscle mass and growth, degenerative/regenerative status, and tissue expression levels.

Firstly, we monitored changes in secreted miRNA during postnatal muscle development, a highly coordinated myogenic differentiation process that occurs globally in all muscle groups. In murine muscle, growth during the perinatal phase is supported by rapid accretion of new nuclei from activated satellite cells (32). Subsequently, the rate of nuclei addition decreases in combination with a reduction in satellite cell numbers per myofibre. After 21 days, the adult level for both the number of nuclei and satellite cells per myofibre is established, with further growth being solely mediated by an increase in the size of the myonuclear domain (32). In agreement with these previous studies, we observed elevated transcript levels of the satellite cell marker *Pax7* in *mdx* and wild-type TA muscle during the first 7 days of life. Subsequently, transcript levels decreased in both genotypes and remained low from 21 PND onward. Strikingly, we also found a strong enrichment of ex-myomiRs during the first week of life in wild-type and pre-symptomatic dystrophic mice (Figure 1), and thus concomitant with the highest rate of satellite activation and myonuclei fusion. In contrast, myofibre growth mediated only by hypertrophy without addition of new nuclei after 21 PND (32), does not seem to be accompanied by elevated ex-myomiR levels in wild-type mice. Importantly, studies in 14-day-old wild-type and dystrophic mice found no evidence of increased membrane permeability (45), therefore arguing against the passive release of ex-myomiRs due to defects in sarcolemma integrity at this age. We therefore conclude that elevation of serum myomiR levels occurs during periods of rapid myofibre growth and maturation, coincident with high rates of myonuclear fusion, and most importantly, in the absence of dystrophic pathology. Our findings demonstrate that tissue-specific miRNAs can be selectively released during neonatal development.

Low level myoinjury caused by downhill exercise induced a biphasic pattern of ex-myomiR release. This finding suggests that myomiRs are released both during the initial myofibre degeneration (immediately after exercise), and during subsequent regeneration (5–7 days post exercise). In the present study, we observed that miR-1 was the most strongly induced miRNA immediately after exercise in both serum and muscle (Figure 2). Previously, similar observations were made for miR-1 in human skeletal muscle after various types of exercise both immediately following the ex ercise as well as up to 10 days later (depending on the mode of exercise and the training status of the individual) (59–61). Interestingly, it has been shown that administration of exogenous miR-1, miR-133, and miR-206 oligonucleotides in rats accelerates muscle regeneration (62). Furthermore, miR-206 is highly expressed in newly regenerated fibres (63), is upregulated in skeletal muscle 7 days after cardiotoxin injury, and miR-206 knockout mice exhibit delayed muscle regeneration (64). Given the importance of myomiRs in regeneration, it is possible that their specific release during postnatal development, and after exercise, constitutes a regulatory signal which promotes myoblast proliferation and differentiation.

Additionally, we monitored the abundance of serum myomiRs during aging (Figure 4) and observed a strong decrease in ex-myomiRs abundance in *mdx* mice at 88 weeks of age compared with adult dystrophic mice, thus suggesting that their abundance in the circulation declines as muscle mass is progressively lost (as is the case with serum CK which also declines in DMD patients with advanced pathology (65)). Importantly, the described reduction in regenerative capacity of aged muscle (66), especially if mediated by changes in the satellite cell niche, could also be related to ex-myomiR levels. Nevertheless, these data highlight a potential complication for the utility of serum myomiRs as biomarkers of DMD disease progression and response to therapy. Previously, myomiRs have been shown to be restored to wild-type levels by dystrophin exon-skipping (21–23,25). However, our results indicate that advanced muscle wasting could also result in return of ex-myomiR abundance to wild-type levels on account of the decline in muscle mass and/or regenerative potential. Similarly, we show that serum myomiR abundance is also affected by exercise. Consequently, ex-myomiR levels in patient serum must be analysed in the context with other clinical information.

While regulation of differentiation by intracellular miRNAs has been extensively studied, regulatory effects of secreted miRNAs are largely unknown. Here we observed a progressive release of myomiRs during terminal differentiation in culture. Differentiation of myoblasts to form multinuclear myotubes in culture has been widely applied as an *in vitro* model of myogenesis. Notably, both primary human myoblasts and C2C12 cultures exhibited a similar, dynamic pattern with a strong induction of myomiR expression and release, especially at the advanced stages of differentiation (Figure 5). Interestingly, this pattern of myomiR release coincides with the generation of multinucleated myocytes that express MHC as these begin to be formed after 3 days and mature continuously by fusing with neighbouring myotubes (Supplementary Material, Figure S4). Importantly, this release occurred with only minimal apoptosis and was specific to myomiRs. In contrast, chemical induction of apoptosis in C2C12 myotubes (Figure 6) resulted in indiscriminate ex-miRNA release, therefore highlighting that although apoptotic cells may contribute to the pool of released miRNAs (55), the observed dramatic increases in ex-myomiR abundance are likely a consequence of selective release (18).

To further characterise the means by which ex-myomiRs are protected from degradation in the extracellular space we used UF-SEC to separate vesicles from protein-complexes. The fractionation of both murine serum and conditioned media derived from C2C12 myotube cultures demonstrated that myomiRs are predominantly present in small protein complexes that elute late during the fractionation process (Figure 7). Conversely, only ∼1% of myomiRs were associated with EVs. These observations are consistent with our previous findings using cruder EV isolation methods and differential sensitivity to Proteinase K-mediated miRNA destabilisation (22). The similarities between cell culture and *in vivo* results suggest that differentiating C2C12 myotubes are a useful model for the investigation of ex-myomiR biology.

We further applied GW4869-mediated suppression of exosome secretion to test the hypothesis that myomiRs might be initially exported in EVs that are subsequently broken down in the circulation (Figure 8). However, the amount of secreted myomiRs remained constant despite successful down-regulation of exosome secretion. This finding argues in favour of either a yet undescribed, non-lipid-based miRNA export process, or direct budding of non-exosomal microvesicles from the cell membrane that is followed by subsequent degradation of these vesicles and liberation of protein-miRNA complexes in the circulation. To this end, it will be important for future work to identify the export machinery for miRNA-protein complexes. If ex-myomiRs do in fact contribute to intracellular communication, specific receptors on recipient cells would similarly need to be identified, as it has yet to be demonstrated that non-lipid encapsulated miRNAs can be taken up by target cells and induce sequence-specific mRNA silencing (67). Currently, there is some limited evidence of ex-miRNAs influencing myogenesis, but only if they are transported in EVs (68,69).

Assuming that myomiRs may participate in intracellular communication, it will be of pivotal importance to determine how many myomiR copies need to be transferred per recipient cell in order to cause a measurable effect on target gene expression. In our study, we aimed to generate more meaningful data by determining absolute copy numbers for both cellular and extracellular miRNAs rather than by simply measuring fold changes. Previous reports state that at least 1,000 miRNA copies per cell are necessary to induce a measurable effect on the mRNA concentration, depending on the target mRNA turnover rate (70,71). Notably, myomiR levels are strongly induced during satellite cell activation and are therefore initially far less abundant in dormant satellite cells than in myotubes (28,72). Therefore, a plausible hypothesis is that differentiating myotubes secrete myomiRs locally and thereby signal to activate neighbouring satellite cells, thus serving to amplify the regenerative response in a paracrine manner. Another possible scenario could be that secreted myomiRs might communicate with other cell types in the muscle niche, such as motor neurons or fibro-adipo progenitors to influence synapse plasticity at the motor end plate or modulate the muscle extracellular milieu respectively. To this end, communication between muscle and neuron has been demonstrated in mouse (73) and drosophila (74), although in both cases signalling was mediated by EVs.

In conclusion, specific increases in serum myomiR abundance during periods of muscle growth, after exercise-induced injury, and concomitant with myogenic differentiation suggest that myomiR release is not exclusively a consequence of leakage from damaged or dystrophic muscle undergoing apoptosis/necrosis. We propose that under some circumstances the presence of myomiRs in the serum may serve a physiological function. Given that the great majority of ex-myomiRs appear to be stabilised in protein complexes, future studies are required to understand the extent to which ex-myomiRs participate in intercellular communication in normal physiology, and how they can be utilised to monitor dystrophic pathology. Despite a plethora of studies investigating the role of miRNAs during stem cell proliferation and progenitor cell differentiation (reviewed in (75)), the influence of secreted miRNAs on these processes is currently not well understood. While our studies have focused on circulating miRNA as relating to myogenesis/muscle regeneration, we propose that ex-miRNAs release is likely to accompany alternative developmental processes and/or cellular differentiation in other lineages, thus comprising a general biological phenomenon that is not exclusive to muscle.

## Materials and Methods

### Animal studies

All animal procedures were carried out in accordance with procedures authorised by the UK home office. Animals used were male C57BL/10 (wild-type, abbreviated as C57) and C57BL/10ScSn-*Dmd^mdx^*/J (abbreviated as *mdx*) mice. For the exercise studies, mice were subjected to 20 min of treadmill exercise (Exer3/6, Columbus instruments, Columbus, OH, USA), running with either a downward incline of 15˚, or on a level surface as according to experimental requirements. Treadmill speed was initially set at 5 m/min and gradually increased to 20 m/min.

Blood was collected from the tail vein for serial measurements. For endpoint measurements animals were sacrificed by escalating CO_2_ concentration and blood harvested from the jugular vein. Animals younger than 14 days were decapitated and blood was harvested from the corpus. In all cases, Microvette CB300 capillary serum tubes (Sarstedt Ltd., Leicester, UK) were used for whole blood collection. Samples were allowed to clot at room temperature for 30 min and then centrifuged at 10,000 *g* for 5 min. Clinical biochemistry assays were performed at the clinical pathology laboratory, Medical Research Council Harwell (Oxford, UK).

### microRNA analysis

We have previously described our methods for the detection and quantification of ex-miRNAs in murine biofluids in detail (34,76). All Reverse Transcriptase quantitative Polymerase Chain Reaction (RT-qPCR) studies were designed to comply with the MIQE guidelines where possible. Briefly, RNA was extracted with TRIzol LS (for biofluid samples) and TRIzol reagent (for cell or tissue samples) according the manufacturer’s protocols (both Life Technologies, Paisley, UK). For serum analysis, the sample volume used for extraction was kept constant within each study (10 μl for serial measurements, 50 μl for end point measurements). Similarly, when conditioned media was analysed, equal volumes (typically 300 μl) of cell culture supernatant were used for RNA isolation. To monitor variation in extraction efficiencies for biofluid samples, 3 μl of a 5 μM synthetic miRNA oligonucleotide, cel-miR-39 (5′-UCACCGGGUGUAAAUCAGCUUG) (IDT, Leuven, Belgium), was added to each sample at the phenol extraction stage. RNA was resuspended in 30 μl of nuclease-free water (Life Technologies).

For miRNA quantification, either 5 µl of the RNA extracted from serum or culture media (or 10 ng of RNA if extracted from cells/tissue) was reversed transcribed using the MicroRNA Reverse Transcription Kit (Life Technologies) and appropriate miRNA specific hairpin RT primer according to the manufacturer’s instructions (assay IDs are listed in Supplementary Material, Table S8). Subsequently, miRNAs were amplified using Small RNA TaqMan assays and TaqMan Gene Expression Master Mix on a Step-One Real-Time PCR instrument (all Life Technologies). For absolute quantification, sample miRNA quantities were compared to a 10 fold dilution series of synthetic miRNA oligonucleotides (IDT) spiked-in at the RT stage. Typical standard curves for all miRNAs are shown in Supplementary Material, Figure S5. This technique enables the comparison of measurements between experiments, and also allows for direct comparison between different miRNA assays. When appropriate, relative quantification was performed using the Pfaffl equation (imputing PCR efficiency values determined empirically by amplification curve analysis using LinRegPCR) (77,78). Sample quantities were normalised to cel-miR-39 levels in the case of biofluids (34), and to miR-16 levels for tissue samples. To obtain serum miRNA concentrations (copy numbers per millilitre), the ratio of input volume used for extraction and RNA resuspension volume was calculated and measured miRNA copy numbers scaled accordingly.

### RT-qPCR

RNA was extracted from cells or tissue using TRIzol reagent and reversed transcribed using the High-Capacity cDNA Reverse Transcription Kit (both Life Technologies) as according to manufacturer’s instructions. Primer and probes used for qPCR were obtained from IDT or Life Technologies and sequences/assay IDs are shown in Supplementary Material, Table S9.

### Cell culture

C2C12 cells were maintained in growth media; Dulbecco’s Modified Eagle’s Media (DMEM) supplemented with 10% fetal bovine serum (FBS) and 1% antibiotics/antimycotics (all Life Technologies) and cultured at 37˚C with 5% CO_2_. C2C12 myoblasts were differentiated in DMEM containing 2% horse serum (HS) for 4-9 days to form multinucleated myotubes. Human primary myoblasts (a kind gift from Helene Fischer (Karolinska Institutet, Stockholm, Sweden)) were obtained from a healthy, 25-year-old, Caucasian male donor with good physical fitness. Cells were cultured in DMEM-F12 (20% FBS, 1% antibiotics/antimycotics) until ∼80% confluency. For induction of differentiation, serum concentrations were initially lowered to 2% HS and then increased after 3 days to 5% HS.

To collect conditioned media for RNA extraction, cells were cultured in differentiation media for 48–72 h. After collection, media was spun at 2,000 *g* for 10 min at 4˚C and subsequently filtered (0.22 μm) to remove cellular debris and apoptotic bodies. To block exosome secretion, C2C12 muscle cells were differentiated for 4 days and then treated for 48 h with GW4869 (Sigma-Aldrich, Dorset, UK) at a final concentration of 10 μM. Subsequently, extracellular vesicles were isolated from the supernatant as described below. To chemically induce apoptosis, cells were differentiated for 6 days and then treated with 1 μM Staurosporine (Abcam, Cambridge, UK) for 24 h. Cell viability was determined by MTS assay using the CellTiter 96 AQueous One Solution Cell Proliferation Assay kit (Promega, Southampton, UK).

### Extracellular vesicle isolation

Extracellular vesicles (EVs) were isolated by either a combinatorial approach of ultrafiltration and size-exclusion chromatography (UF-SEC) as described previously (57) or by ultracentrifugation (UC). To isolate EVs from conditioned media, cells were cultured in pre-spun media which contains serum that has been pre-cleared of serum EVs (120,000 *g*, 70 min). For EV isolation by UF-SEC, conditioned media or serum was spun at 2,000 *g* for 10 min at 4˚C, filtered (0.22 μm) and subsequently concentrated on a 10 kDa cut-off Amicon Ultra-15 spin filter (EMD Millipore, Watford, UK) at 3,500 *g* for 15 min. Filters were washed with PBS and the combined retentate was loaded onto a HiPrep 16/60 Sephacryl S-400 (GE Healthcare, Pollards Wood, UK) connected to an ÄKTA prime (GE Healthcare) equipped with a UV flow cell where fixed volume (2 ml) fractions were collected. Based on UV 280nm absorbance values (indicative of protein content), the collected fractions were grouped into 4-6 new fractions. These fractions were pooled and re-concentrated using the 10 kDa cut-off filter (EMD Millipore) to a final volume of ∼200 μl. For EV isolation by UC, conditioned media was prepared as described above and ultracentrifuged twice at 120,000 *g* for 70 min and pellets were washed with phosphate-buffered saline (PBS) between the two spins to minimise protein contamination. EV-pellets were re-suspended in a final volume of 50 μl in PBS.

### Nanoparticle tracking analysis

Nanoparticle tracking analysis (NTA) was performed to determine particle counts and size with a NanoSight NS500 instrument (Malvern Instruments Ltd, Malvern, UK) equipped with the NTA 2.3 analytical software. Samples were diluted in PBS to achieve a particle count of between 2 × 10^8^ per ml and 2 × 10^9^ per ml. Three 30s videos for each sample were recorded using the script control function and measurements were then analysed using the batch process facility.

### Immunofluorescence and trypan blue exclusion assay

To detect apoptosis, the FITC Annexin V Apoptosis Detection Kit I (BD Pharmingen, Plymouth, UK) was used for fluorescence staining with minor alterations to the manufacturer’s protocol. In brief, cells were washed in 1x Binding Buffer and then stained for 30 min with propidium iodide, Annexin V-FITC (both 1:30) and Hoechst (1:1000).

Trypan Blue was also used to assess cell viability by dye exclusion assay. In brief, cells were incubated with 0.4% Trypan Blue Solution (Sigma-Aldrich) for 2 min and analysed for dye uptake with the EVOS FL cell imaging system (Life Technologies).

### Western blot

Samples were diluted 1:1 with 2× Laemmli sample buffer (Bio-Rad, Hemel Hempstead, UK) containing 5% β-mercaptoethanol and heated at 100 °C for 10 min. Western blot was performed using the following primary antibodies; anti-CD9 (ab92726), anti-ALIX (PDC6IP) (ab117600) and anti-TSG101 (ab30871); all at 1:1,000 dilution (all Abcam). For detection, secondary antibodies conjugated to infrared dyes (anti-mouse IgG IRDye800 (926-32210) for detection of ALIX (1:10,000); anti-rabbit IgG 680RD (926-68071) for detecting CD9 and TSG101 (1:10,000)) and LI-COR Odyssey CLx infrared imaging system were used (all LI-COR, Cambridge, UK).

### ELISA

CD63 ELISA (ABIN1572929, Antibodies Online, Aachen, Germany) was performed according to manufacturer’s instructions. Each fraction was diluted to fall within the linear range of the assay. Sample concentrations were extrapolated with GraphPad Prism 5 (GraphPad Software Inc, La Jolla, CA) using fourth-order polynomial data fit of the standard curves. CD63 quantities were normalised to total protein amounts as determined by micro BCA assay (Thermo Fisher Scientific, Bicester, UK).

### Statistical analysis

Statistical analyses were carried out utilising GraphPad Prism 5. Comparisons between two groups were tested using a two-sided *t*-test. One-way analysis of variance (ANOVA) and Bonferroni correction *post hoc* test were performed for comparisons of one variable in more than two groups. If the influence of two independent variables was tested, two-way ANOVA with Bonferroni post testing was applied. Differences were considered significant at *P*-values less than 0.05. Pearson correlation analysis was performed with GraphPad Prism 5.

## Supplementary Material

Supplementary Material is available at *HMG* online.

*Conflict of Interest statement*. None declared.

## Funding

This work was supported by the Medical Research Council (MRC doctoral training award 1371292 – ACS and MRC Centenary Early Career Award – TCR). The funders had no role in study design, data collection and analysis, decision to publish, or preparation of the manuscript. Funding to pay the Open Access publication charges for this article was provided by Oxford’s RCUK Open Access Block Grant. YL was supported by the Agency for Science, Technology and Research (A*STAR, Singapore). IM was supported by Estonian Research Council (Personal Research Grant PUT618). SELA was supported by the Swedish Research Council. JEM was supported by the National Institute for Health Research Biomedical Research Centre at Great Ormond Street Hospital for Children NHS Foundation Trust and University College London and by the Great Ormond Street Hospital Children’s Charity.
